# Induction of heme oxygenase-1 protects against nutritional fibrosing steatohepatitis in mice

**DOI:** 10.1186/1476-511X-10-31

**Published:** 2011-02-12

**Authors:** Rong Qi Wang, Yue Min Nan, Wen Juan Wu, Ling Bo Kong, Fang Han, Su Xian Zhao, Li Kong, Jun Yu

**Affiliations:** 1 Department of Traditional and Western Medical Hepatology, Third Hospital of Hebei Medical University, Shijiazhuang, China; 2 Institute of Digestive Disease and Department of Medicine and Therapeutics, Li Ka Shing Institute of Health Sciences, The Chinese University of Hong Kong, Hong Kong

## Abstract

**Background:**

Heme oxygenase-1 (HO-1), an antioxidant defense enzyme, has been shown to protect against oxidant-induced liver injury. However, its role on liver fibrosis remains unclear. This study aims to elucidate the effect and the mechanism of HO-1 in nutritional fibrosing steatohepatitis in mice.

**Methods:**

Male C57BL/6J mice were fed with a methionine-choline deficient (MCD) diet for eight weeks to induce hepatic fibrosis. HO-1 chemical inducer (hemin), HO-1 chemical inhibitor zinc protoporphyrin IX (ZnPP-IX) and/or adenovirus carrying HO-1 gene (Ad-HO-1) were administered to mice, respectively. Liver injury was assessed by serum ALT, AST levels and histological examination; hepatic lipid peroxides levels were determined; the expression levels of several fibrogenic related genes were assayed by real-time quantitative PCR and Western blot.

**Results:**

MCD feeding mice showed progressive hepatic injury including hepatic steatosis, inflammatory infiltration and fibrosis. Induction of HO-1 by hemin or Ad-HO-1 significantly attenuated the severity of liver injury. This effect was associated with the up-regulation of HO-1, reduction of hepatic lipid peroxides levels, down-regulation of inflammatory factors tumor necrosis factor-alpha, interleukin-6 and suppressor of cytokine signaling-1 as well as the pro-fibrotic genes alpha-smooth muscle actin, transforming growth factor-β1, matrix metallopeptidase-2 and matrix metallopeptidase-9. A contrary effect was observed in mice treated with ZnPP-IX.

**Conclusions:**

The present study provided the evidence for the protective role of HO-1 in ameliorating MCD diet-induced fibrosing steatohepatitis. Modulation of HO-1 expression might serve as a therapeutic approach for fibrotic steatohepatitis.

## Introduction

Non-alcoholic steatohepatitis (NASH) represents a major medical problem worldwide and has been recognized as a leading cause of liver fibrosis [1]. In patients with NASH, approximately 74% and 30% progresses to fibrosis and cirrhosis [2,3]. Although the progression of fibrosis is the critical pathophysiological feature of NASH, the mechanism of fibrogenesis in the presence of steatohepatitis has not been well elucidated and specific therapies are lacking. Reactive oxygen species (ROS) and oxidative stress are the major causes of liver damage and involved in the development of hepatic fibrosis by inducing hepatic stellate cells (HSC) proliferation and collagen synthesis [4]. Oxidative stress also promotes pro-inflammatory cytokines secretion, such as tumor necrosis factor-alpha (TNF-α) and interleukin-6 (IL-6), which are able to stimulate collagen synthesis [5,6]. Suppressor of cytokine signaling-1 (SOCS-1) was an important inhibitor of inflammation. It inhibits the signaling pathway of many cytokines such as IL-2, IL-6, TNF-α, IFN-α and IFN-γ [7] and influences the inflammation and fibrosis of liver [8]. It is known that activation of HSCs is a critical step in hepatic fibrogenesis. HSCs activation is regulated by cytokines and ROS released by damaged hepatocytes and activated Kupffer cells and even HSCs themselves [9,10]. Once activated, HSCs transform into proliferative, fibrogenic, and contractile myofibroblasts and secrete excessive extracellular matrix (ECM) components [11]. ECM, which is a reservoir for growth factors and matrix metalloproteinases (MMPs), has been shown to actively participate in the progression of liver fibrosis by providing survival factors for activated HSCs [12]. The excess deposition of ECM can disrupt the normal architecture of the liver resulting in pathophysiological damage to the liver. Therefore, suppression oxidative stress, inhibition of HSC activation and its related subsequent events, such as increased production of ECM components and fibrogenic cytokines, could be a therapeutic approach for liver fibrosis.

Heme oxygenase (HO) is the rate-limiting enzyme for heme degradation in mammals. It can metabolize the oxidative degradation of heme into equimolar amounts of biliverdin/bilirubin, carbon monoxide (CO), and free iron [13]. Three isoforms of HO have been characterized: the stress-inducible protein HO-1, widely expressed at a low level in mammalian tissues and can be induced by a variety of stimuli, including heme, heavy metals, cytokines, endotoxins and oxidative stress [14]; the constitutive HO-2, expressed mainly in the brain and testis and is unresponsive to any of the known HO-1 inducers [15]; and the HO-3, who shares about 90% amino acid homology with HO-2, has a very low enzyme activity and constitutively expressed in the liver in rats [16]. HO-1 occurs at low to undetectable levels in live and expresses mainly in Kupffer cells under basal conditions, but it undergoes a rapid transcriptional activation and expresses in both Kupffer cells and hepatocytes as response to noxious stimuli [17]. HO-1 induction is considered to be an adaptive cellular response to survive exposure to environmental stresses. Although the function of this enzyme is not completely understood, increasing evidence suggests that HO-1 plays a vital role in many aspects, such as suppression of oxidative stress, inflammation and cell proliferation, microcirculation improvement and regulation of cytokine expression in various pathological conditions [18-20]. However, there are few investigations regarding the protective effect of HO-1 on liver fibrosis. Importantly, no evidence is available for whether the expression of HO-1 contributes to the pathophysiological changes in the development of nutritional fibrosing steatohepatitis. Thus, the present study was designed to investigate the effects of HO-1 expression on liver fibrosis induced by MCD diet in mice.

## Materials and methods

### Animals and treatments

Eight-week-old male C57BL/6J mice were obtained from the Experimental Animal Center of the Chinese Academy of Medical Sciences, and were bred in a temperature-controlled animal facility with a 12-h light-dark cycle. The animals had free access to water and were allowed to adapt to their food and environment for 1 week before the start of the experiment. Mice were randomly divided into 7 groups (6 mice per group): 1) MCD group, mice fed methionine-choline deficient diet (ICN, Aurora, Ohio, USA); 2) control group, mice fed MCD diet supplemented with choline bitartate(2 g/kg) and DL-methionine (3 g/kg) (ICN, Aurora, Ohio); 3) MCD+hemin group, mice fed MCD diet administered with HO-1 chemical inducer hemin (30 μmol/kg) by intraperitoneal (i.p.) injections three times per week; 4) MCD+ZnPP group, mice fed MCD diet administered with HO-1 inhibitor ZnPP-IX (20 μmol/kg) by i.p. injections three times per week; 5) MCD+Ad-GFP group, mice fed MCD diet administered with adenovirus encoding green fluorescent protein (2.5 × 10^8 ^Plaque-forming units (pfu) by i.p. injections two times per week; 6) MCD+Ad-HO-1 group, mice fed MCD diet administered with adenovirus encoding the full-length mouse HO-1(2.5 × 10^8 ^pfu) (Ad-HO-1) by i.p. injections two times per week; 7) MCD+hemin+Ad-HO-1 group, mice fed MCD diet administered with hemin and Ad-HO-1. The duration of the experiment is up to 8 weeks. During the experiments, the body-weight and rate of diet consumption were recorded. At the end of the experiment, all of the animals were sacrificed after overnight fasting. Blood samples were collected from the femoral artery for biochemical analysis. Livers were weighed and fixed in 10% formalin for histological analysis, or snap-frozen in liquid nitrogen followed by storage at -80°C freezer until required. All the protocols and procedures were carried out in accordance with the guidelines of the Hebei Committee for Care and Use of Laboratory Animals and were approved by the Animal Experimentation Ethics Committee of the Hebei Medical University.

### Construction of recombinant adenoviru

A recombinant adenovirus containing the entire coding sequence of mouse HO-1 (Ad-HO-1) and control adenovirus encoding green fluorescent protein (Ad-GFP) were purchased from Tianjing Saier Biochemistry limited company (Tianjing, China). Adenovirus was propagated, isolated in human embryonic kidney 293 (HEK 293) cells and purified with Adeno-X Virus Purification kit (Clontech, Mountain View, CA, USA). Titer of the viral solution was determined by Adeno-X Rapid Titer kit (Clontech). The virus was stored at -80°C until use. Mice were given intraperitoneal injection of Ad-HO-1 or Ad-GFP at an amount of 2.5 × 10^8 ^pfu suspended in 100 μl phosphate-buffered saline two times per week.

### Measurement of serum ALT and AST

Serum alanine aminotransferase (ALT) and aspartate aminotransferase (AST) levels were measured by the enzymatic kinetic method using an automatic biochemical analyzer (Olympus UA2700, Japan) according to the manufacturer's instructions.

### Histologic evaluation

Formalin-fixed liver was embedded in paraffin and 4 μm sections were stained with Haematoxylin and eosin and Masson-trichrome. Two experienced hepatopathologist independently evaluated the slides and assigned a score for steatosis and fibrosis as described previously [21]. The stage of fibrosis was assessed using a 4-point scale (1, mild/moderate zone 3 perisinusoidal fibrosis, or portal fibrosis only; 2, zone 3 and portal/periportal fibrosis; 3, bridging fibrosis; and 4, cirrhosis).

### Malondialdehyde (MDA) assay

The extent of lipid peroxidation in the liver homogenate was evaluated by measuring the concentration of the thiobarbituric acid-reactive product, malondialdehyde (MDA), using a thiobarbituric acid-reactive substrances (TBARS) assay kit (Cell Biolabs, Inc. San Diego, CA) [22].

### Hydroxyproline Analysis

The degree of liver fibrosis was determined by the measurement of hydroxyproline content in livers (Jiancheng Bioengineering, Nanjing, China), which could represent the total amount of collagens in livers. The experiment was performed as previously described [23]. The hydroxyproline levels in the hydrolysates were measured by detection of the absorbance at 550 nm. A standard curve of samples with known quantities of hydroxyproline was generated for each assay. The absorbances of unknown samples were compared to the standard curve, hydroxyproline content was calculated and expressed as microgram hydroxyproline/milligram liver. Each sample was assayed in triplicate.

### Quantitation of hepatic messenger RNA expression levels

Total RNA was extracted from the frozen liver tissues by using RNA Trizol reagent (Invitrogen, Carlsbad, CA). Five microgram of total RNA for each sample was reverse transcribed into complementary DNA (cDNA), the cDNA was diluted 1/100 and 5 μl were used as a template per PCR reaction. The quantitative real-time PCR was performed on an ABI PRISM 7300 PCR System (Applied Biosystems, Foster City, CA) using Syber Green I GoTaq^® ^qPCR Master Mix (Promega BioSciences. Sunnyvale, CA). Expression levels of the target genes generated standard curves were normalized against an endogenous reference gene glyceraldehydes 3-phosphate dehydrogenase (GAPDH). For each sample and each gene, PCR were carried out in duplicate and repeated twice. The relative expression of target genes were obtained by the software SDS v1.3.2 attached with the PCR machine. Levels of mRNA expression of TNF-α, IL-6 and SOCS1 were also determined by reverse transcription semi-quantitative polymerase chain reaction (RT-PCR) in a 25 μl reaction volume using Promega Green Master Mix (Promega, San Luis Obispo, CA) on the ABI Prism 2720 (Applied Biosystems, Foster City, CA). The specific primer sequences were listed in Table 1.

**Table 1:** Primers for real-time quantitative PCR analysis

Gene	Product length	Primer sequences
HO-1	427bp	F 5'-AACAAGCAGAACCCAGTCTATG-3'
		R 5'-TGAGCAGGAAGGCGGTCTTA-3'
TNF-α	300bp	F 5'-GGCAGGTCTACTTTGGAGTCATTGC-3'
		R 5'-ACATTCGAGGCTCCAGTGAATTCGG-3'
IL-6	159bp	F 5'-AGT TGC CTT CTT GGG ACT GA-3'
		R 5'-TCC ACG ATT TCC CAG AGA AC-3'
α-SMA	160bp	F 5'-CTGACAGAGGCACCACTGAA-3'
		R 5'-CATCTCCAGAGTCCAGCACA-3'
TGF-β1	272bp	F 5'-CAACGCCATCTATGAGAAAACC-3'
		R 5'-ACTGCCGTACAACTCCAGTGAC-3'
MMP-2	203bp	F 5'-CACACCAGGTGAAGGATGTG-3'
		R 5'-AGGGCTGCATTGCAAATATC-3'
MMP-9	67bp	F 5'-CAGTATCTGTATGGTCGTGGCT-3'
		R 5'-TTCAGTTGTGGTGGTGGCT-3'
SOCS1	123bp	F 5'-TCCTCGTCCTCGTCTTCGT-3'
		R 5'-TAATCGGAGTGGGAGCGGA-3'
GAPDH	450bp	F 5'-ACCACAGTCCATGCCATCAC-3'
		R 5'-TCCACCACCCTGTTGCTG-3'

Abbreviations: HO-1, heme oxygenase-1; TNF-α, tumor necrosis factor-alpha; IL-6, interleukin-6; α-SMA, α-smooth muscle actin; TGF-β1, transforming growth factor beta 1; MMP-2, matrix metallopeptidase-2; MMP-9, matrix metallopeptidase-9; SOCS1, suppressor of cytokine signaling-1; GAPDH, glyceraldehyde 3-phosphate dehydrogenase.

### Western blotting analysis of hepatic protein expression

Total protein was extracted and concentration was measured by the Bradford method (DC protein assay; Bio-Rad, Hercules, CA). Equal amounts of protein (100 μg/well) were loaded onto 10% SDS-PAGE for each sample and proteins were transferred onto equilibrated polyvinylidene difluoride membranes (Amersham Biosciences, Buckinghamshire, UK) by electroblotting. Membranes were incubated overnight at 4°C with primary antibodies (Santa Cruz Biotechnology, Santa Cruz, CA). After incubation with the secondary antibody, proteins were detected by enhanced chemiluminescence (ECL, Amersham Corporation). The amount of protein expression was corrected by the amount of β-actin in the same sample and the bands were quantified by scanning densitometry using the digital Kodak Gel Logic 200 (Carestream Molecular Imaging, USA).

### Statistical analysis

All data are expressed as mean ± standard deviation (SD). Statistical analysis was carried out by one-way analysis of variance (ANOVA) and the Student-Newman- Keuls test for evaluating differences between groups using SPSS 13.0 (v.13.0; SPSS Inc., Chicago, III, USA). A *P*-value of less than 0.05 was considered statistically significant.

## Results

### Effect of HO-1 on MCD diet-induced fibrosing steatohepatitis

**Figure 1:**
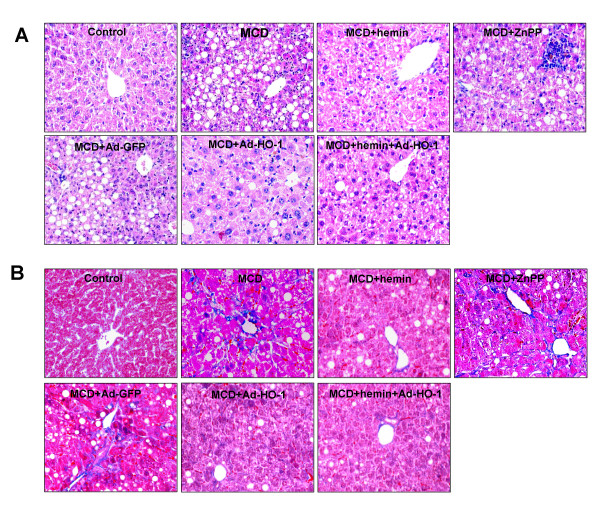
**Histopathological changes of liver sections of mice under various treatment conditions**. Hematoxylin and eosin stained (A) and Masson trichromatism stained (B) liver sections from mice livers (Original magnification, ×200).

**Figure 2:**
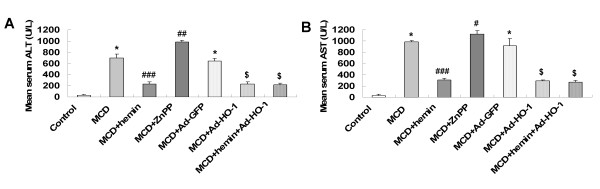
**Effects of the MCD diet and treatment with hemin and/or Ad-HO-1 or ZnPP-IX on: (A) Serum alanine aminotransferase (ALT); (B) Serum aspartate aminotransferase (AST)**. Data are expressed as mean ± SD (n = 6 per group). *P < 0.001, compared with control group; ^#^*P *< 0.05, ^##^*P *< 0.01, ^###^*P *< 0.001, compared with MCD group; ^$^*P *< 0.001, compared with MCD+Ad-GFP group.

**Figure 3:**
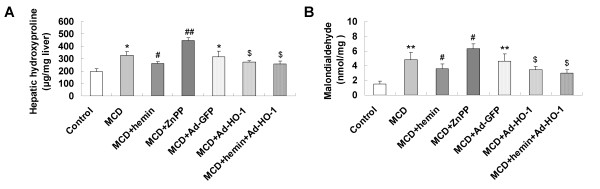
**Effects of MCD diet and treatment with hemin and/or Ad-HO-1 or ZnPP-IX on: (A) liver hydroxyproline content**; **(B) hepatic malondialdehyde (MDA) content.** Data are expressed as mean ± SD (n = 6 per group). *P < 0.01, **P < 0.001, compared with control group; ^#^*P *< 0.05, ^##^*P *< 0.01, compared with MCD group; ^$^*P *< 0.05, compared with MCD+Ad-GFP group.

Mice fed with MCD diet for 8 weeks exhibited disordered lobule structure, macrosteatosis in Zone 3, spot or focal hepatocyte necrosis, inflammatory infiltration (Figure 1A) and portal and perisinusoidal fibrosis (Figure 1B). Treatment with HO-1 inducer hemin or Ad-HO-1 markedly reduced the severity of hepatic steatosis, inflammatory infiltration (Figure 1A) and fibrosis (Figure 1B), which was associated with a prominent decrease in serum ALT (Figure 2A), AST (Figure 2B) levels and hepatic hydroxyproline content (Figure 3A). Co-administration of hemin and Ad-HO-1 gave similar effect without further improvement. In contrast, hepatic steatosis, inflammatory infiltration and fibrosis were aggravated (Figure 1), the serum transaminases levels (Figure 2) and hepatic hydroxyproline content (Figure 3A) were further elevated in mice treated with ZnPP-IX than those fed MCD diet only. These showed that HO-1 induction protected mice from MCD diet-induced liver injury.

### Effect of HO-1 induction on liver malondialdehyde

Malondialdehyde (MDA) was analyzed as an indicator of lipid peroxidation. Mice intake of the MCD diet resulted in a significant increase in hepatic MDA content compared with that of the control mice, indicating hepatic oxidative stress. An impressive reduction of hepatic MDA was noted after hemin treatment for 8 weeks compared to MCD-treated mice. A similar effect was observed by Ad-HO-1 gene transfer compared to mice administered Ad-GFP. The combination of hemin and Ad-HO-1 failed to show an additive effect on suppressing MDA concentrations. However, the levels of hepatic MDA was significantly increased in mice treated with ZnPP-IX than those fed MCD diet only (Figure 3B). Measurement of hepatic MDA revealed that HO-1 induction ameliorated oxidative injury induced by MCD feeding.

### Induction of HO-1 by hemin and/or Ad-HO-1

**Figure 4:**
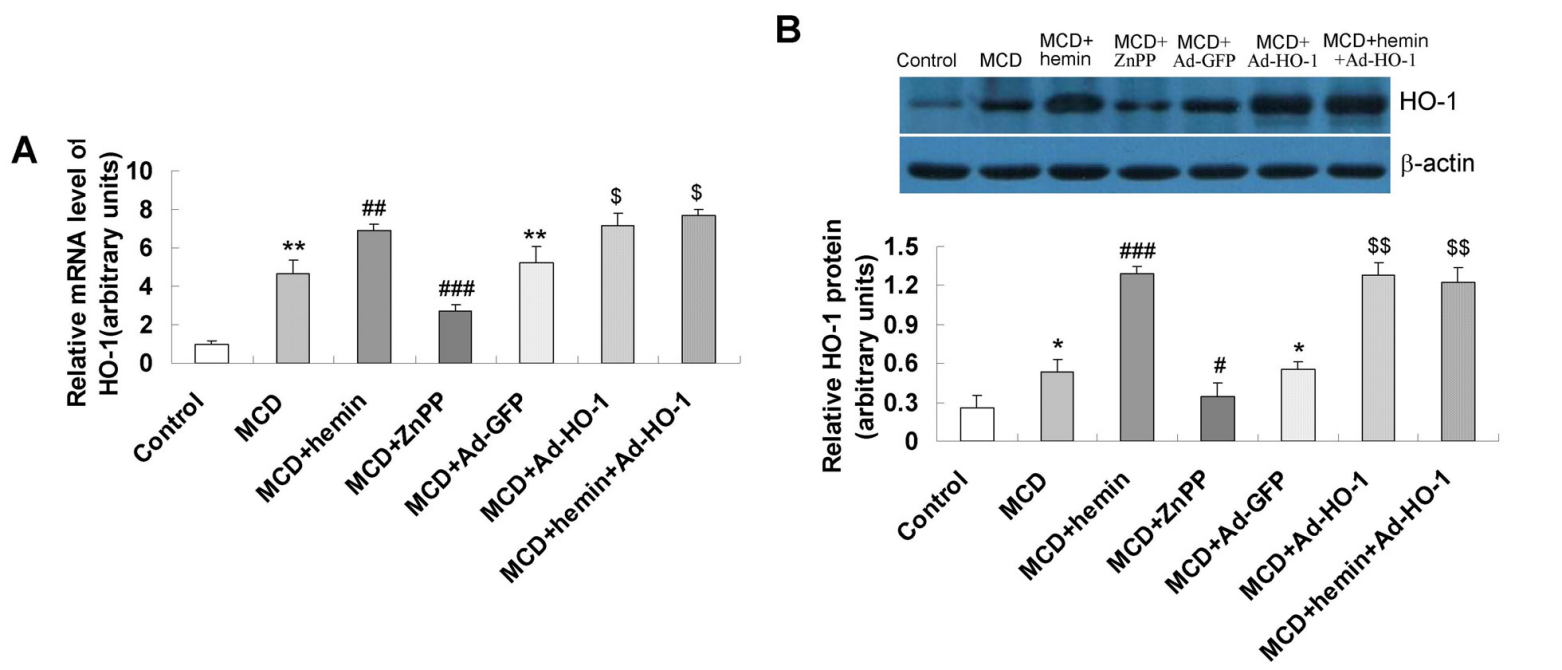
**Effects of hemin and/or Ad-HO-1 on hepatic HO-1 mRNA and protein expression in the livers of mice**. (A) Hepatic HO-1 mRNA, determined by real-time quantitative PCR; (B) HO-1 protein levels, detected by Western blot. Data are expressed as the mean ± SD (n = 6 per group). *P < 0.01, **P < 0.001, compared with control group; ^#^*P *< 0.05, ^##^*P *< 0.01, ^###^*P *< 0.001, compared with MCD group; ^$^*P *< 0.01, ^$$^*P *< 0.001, compared with MCD+ Ad-GFP group.

The mRNA and protein expression of HO-1 was barely detectable in mice fed control diet, increased in mice fed the MCD diet and markedly increased in mice treated with hemin or Ad-HO-1 (Figure 4), which paralleled the improvement in histological severity of steatohepatitis. Co-administration of hemin and Ad-HO-1 had no better effect on up-regulation of HO-1 expression. Conversely, in ZnPP-IX treatment mice, hepatic mRNA and protein expression of HO-1 was inhibited in company with a pronounced liver injury (Figure 4).

### Effect of HO-1 on the expression of inflammatory genes

**Figure 5:**
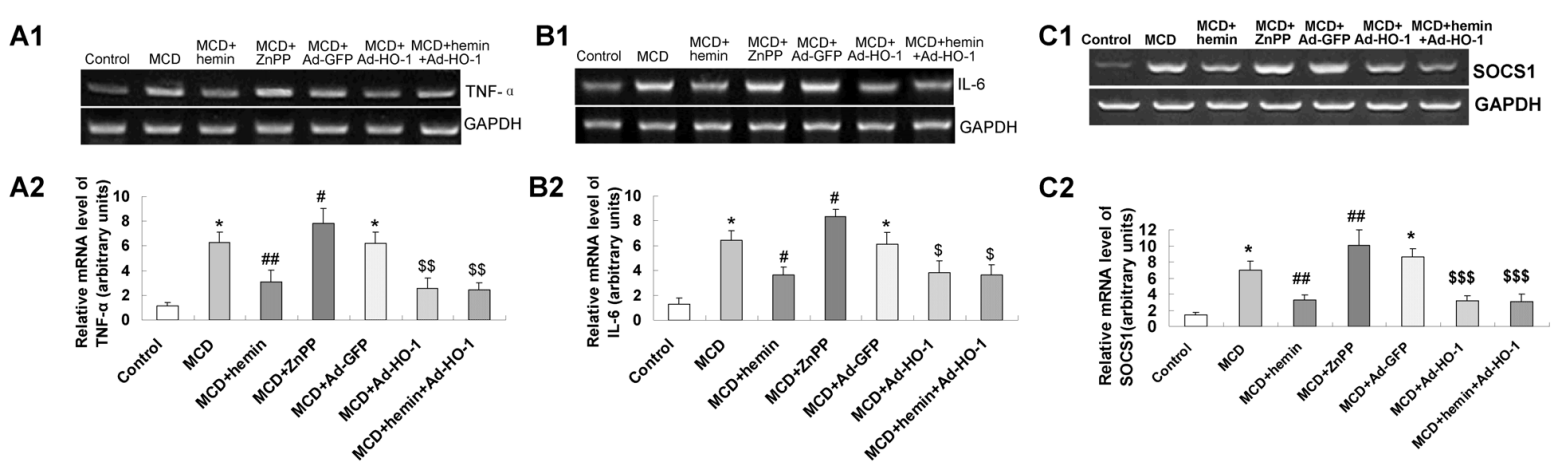
**Effects of HO-1 on hepatic inflammatory factors in MCD diet-induced fibrosing steatohepatitis**. (A) Hepatic TNF-α mRNA levels were determined by (A1) RT-PCR and (A2) real-time quantitative PCR; (B) Hepatic interleukin-6 (IL-6) mRNA levels, determined by (B1) RT-PCR and (B2) real-time quantitative PCR; (C) Hepatic SOCS1 mRNA levels were determined by (C1) RT-PCR and (C2) real-time quantitative PCR. Data are expressed as the mean ± SD (n = 6 per group). *P < 0.001, compared with control group; ^#^*P *< 0.05, ^##^*P *< 0.01, compared with MCD group; ^$^*P *< 0.05, ^$$^*P *< 0.01, ^$$$^*P *< 0.001, compared with MCD+ Ad-GFP group.

To seek an explanation for the decreased serum transaminases and ameliorated liver histology by inducing HO-1, we investigated hepatic messenger RNA (mRNA) expression levels of inflammatory genes TNF-α, IL-6 and SOCS1. Relative to control mice, Hepatic TNF-α, IL-6 and SOCS1 genes were up-regulated in MCD diet-fed mice (Figure 5). This induction was significantly blunted by the treatment with hemin or Ad-HO-1. The combination of hemin and Ad-HO-1 caused similar effects on preventing the TNF-α, IL-6 and SOCS1 mRNA expression. Whereas, supplement with ZnPP-IX led to a further increase of TNF-α, IL-6 and SOCS1 expression compared with mice fed MCD diet only (Figure 5).

### Effect of HO-1 on the expression of fibrosis related genes

**Figure 6:**
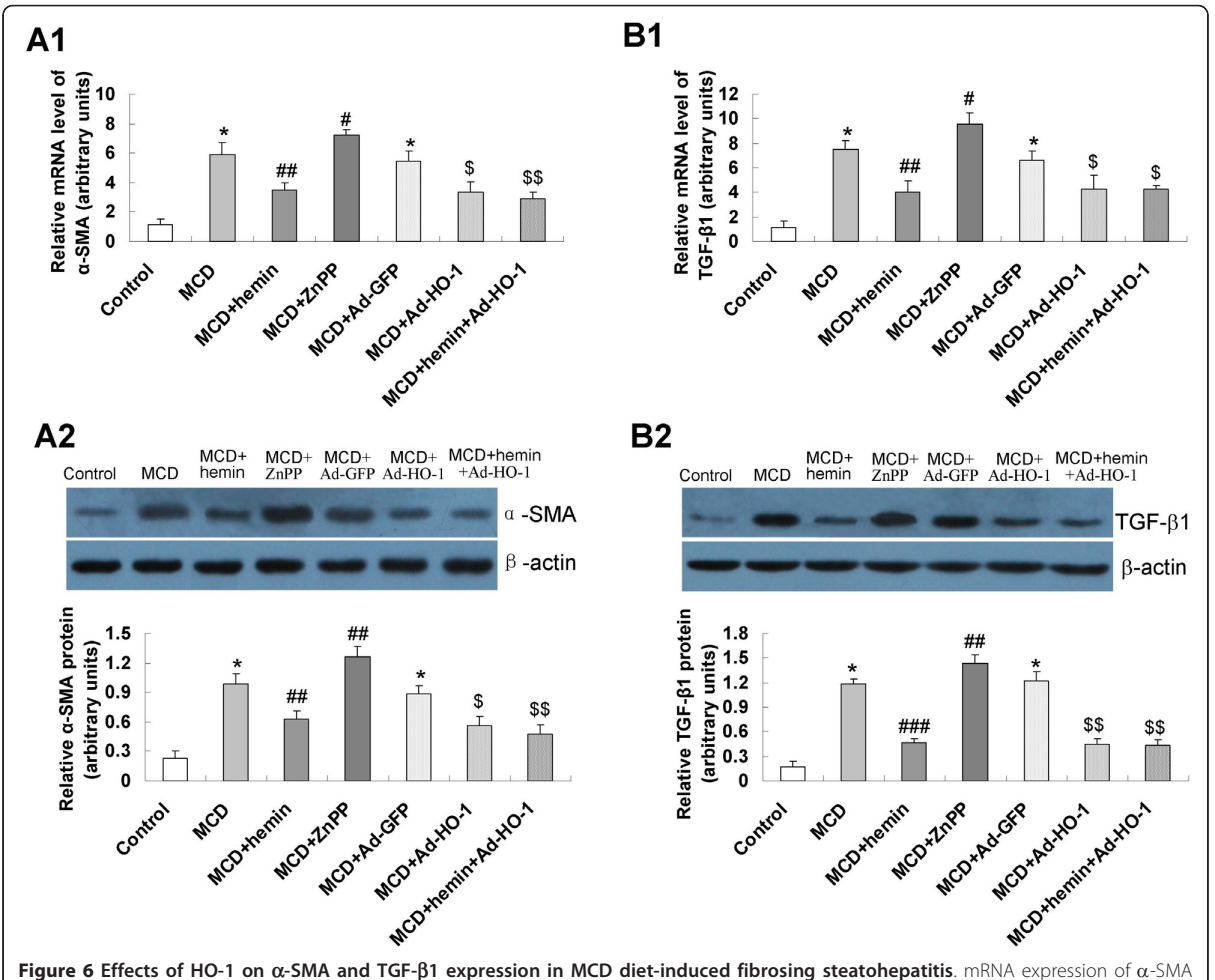
**Effects of HO-1 on α-SMA and TGF-β1 expression in MCD diet-induced fibrosing steatohepatitis**. mRNA expression of α-SMA (A1) and TGF-β1 (B1) were determined by real-time quantitative PCR; protein levels of α-SMA (A2) and TGF-β1 (B2) were detected by Western blot. Data are expressed as the mean ± SD (n = 6 per group). *P < 0.001, compared with the control group; ^#^*P *< 0.05, ^##^*P *< 0.01, ^###^*P *< 0.001, compared with MCD group; ^$^*P *< 0.01, ^$$^*P *< 0.001, compared with MCD+Ad-GFP group.

**Figure 7:**
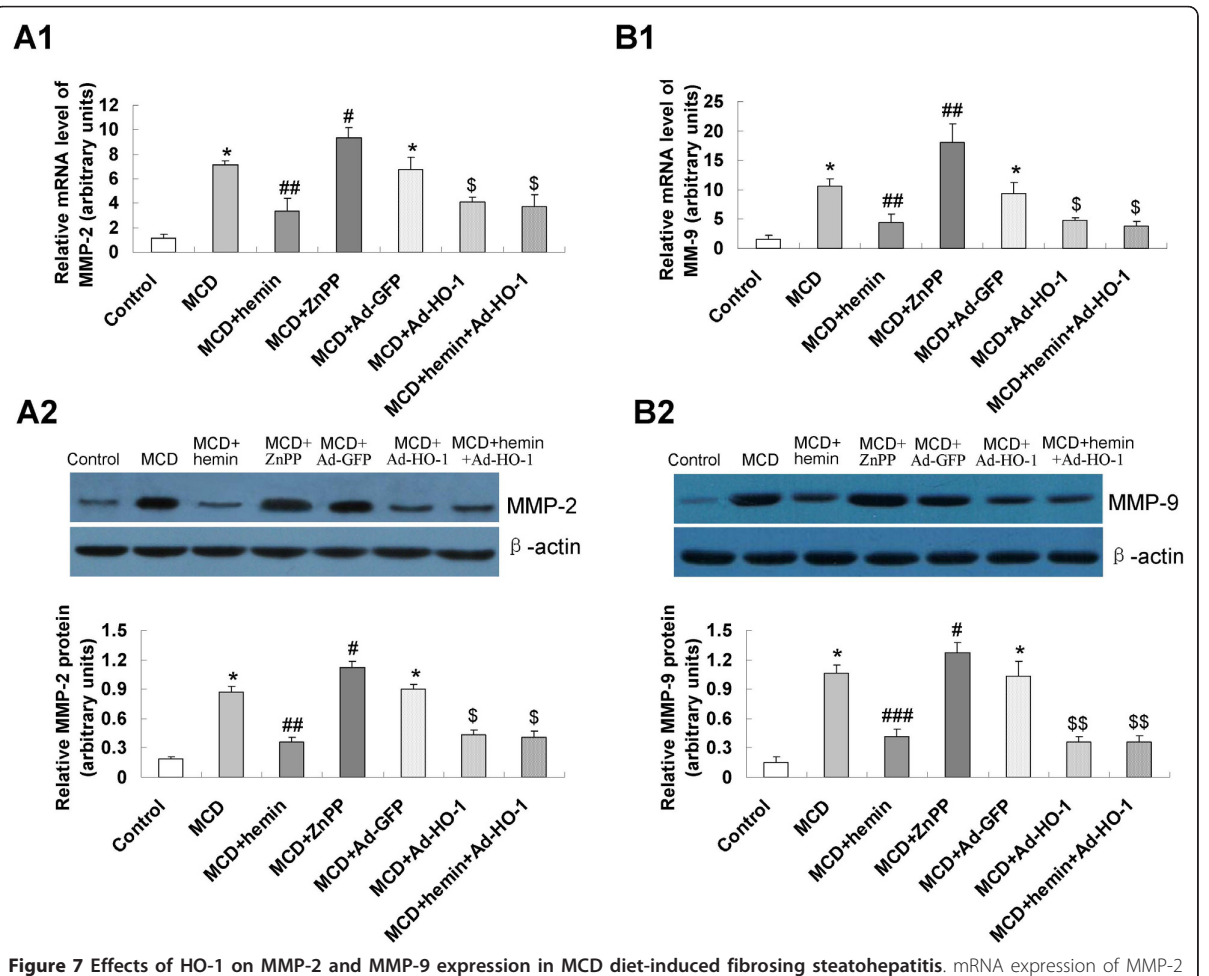
**Effects of HO-1 on MMP-2 and MMP-9 expression in MCD diet-induced fibrosing steatohepatitis**. mRNA expression of MMP-2 (A1) and MMP-9 (B1) were determined by real-time quantitative PCR; protein levels of MMP-2 (A2) and MMP-9 (B2) were detected by Western blot. Data are expressed as the mean ± SD (n = 6 per group). *P < 0.001, compared with the control group; ^#^*P *< 0.05, ^##^*P *< 0.01, ^###^*P *< 0.001, compared with MCD group; ^$^*P *< 0.01, ^$$^*P *< 0.001, compared with MCD+Ad-GFP group.

To evaluate the mechanisms of the effect of HO-1 induction on fibrosing steatohepatitis, we assessed the hepatic expression level of fibrosis related genes. Mice fed a MCD diet showed enhanced expression of hepatic mRNA and protein of alpha-smooth muscle actin (α-SMA) (Figure 6A), transforming growth factor-β1 (TGF-β1) (Figure 6B), matrix metallopeptidase-2 (MMP-2) (Figure 7A) and matrix metallopeptidase-9 (MMP-9) (Figure 7B), which was significantly blunted by the treatment with hemin or Ad-HO-1. A similar effect was observed in hemin plus Ad-HO-1 group. Conversely, hepatic mRNA and protein expressions of α-SMA, TGF-β1 (Figure 6), MMP-2 and MMP-9 (Figure 7) were further up-regulated by ZnPP-IX treatment as compared to MCD group.

## Discussion

With use of the MCD dietary model, mice consistently develop a form of fibrosing steatohepatitis. The resulting characteristic pathology of steatosis mixed inflammatory cell infiltrate, hepatocyte necrosis, and appreciable intraparenchymal pericellular fibrosis mimics the finding in human NASH [24]. In addition, MCD diet caused significant increase in serum ALT and AST levels, hepatic hydroxyproline concentrations in C57BL/6J mice. Taking ALT and AST as reliable indicators of liver inflammatory injury and hepatic hydroxyproline as the amount of collagen fibers, we found that hepatic inflammation and fibrosis were alleviated via hemin or Ad-HO-1 treatment. However, the combination of Ad-HO-1 and hemin did not further ameliorate the liver injury. On the other hand, treatment with HO-1 chemical inhibitor ZnPP-IX down-regulated the expression of HO-1 and augmented the liver injury. Wei et al. [25] found that HO-1 was expressed at a high level in the early or initiation stage of cirrhosis, and AST kept at a marked low level [26]. These indicated that HO-1 might play an important role in prevention of hepatic inflammation and fibrosis.

Oxidative stress is an essential "second hit" in the pathogenesis of NASH, because increased free radicals can cause lipid peroxidation which induces inflammatory response and stellate cells activation, and further leads to collagen synthesis and fibrogenesis [27]. The antioxidant defense mechanism was altered by accumulation of the products derived from oxidative damage such as malondialdehyde (MDA) [28]. MDA can produce ozone, hydrogen peroxide and other active oxygen species, cause peroxidation and denaturation of cellular membranes [29], which impact on the HSCs to induce a fibrogenic response [6]. In the present study, we found that up-regulation of HO-1 by hemin or Ad-HO-1 could decrease the hepatic MDA levels, improve the hepatic oxidative damage in MCD diet induced fibrosing steatohepatitis. Xue and colleagues [30] found that HO-1 overexpression protected hepatic ischemia/reperfusion injury in cirrhotic rats by lowering serum MDA and increasing serum manganese superoxide dismutase (MnSOD). The protective effects of HO-1 may be due to its antioxidative product biliverdin/bilirubin, which can scavenge peroxide radicals and inhibit lipid peroxidation [14]. Therefore, we speculate that high HO-1 expression induced by hemin or Ad-HO-1 appears to be a good choice for preventing the oxidative injury in fibrosing steatohepatitis.

Normally, oxidative injury leads to damage of hepatocytes, which is followed by activation of Kupffer cells and result in production of a number of inflammatory cytokines, including TNF-α and IL-6 [12]. This regulation might explain the enhancement of hepatic TNF-α and IL-6 mRNA expression in MCD fed mice. In response to proinflammatory cytokines of TNF-α and IL-6, the mRNA expression of SOCS1 is also increased in MCD fed mice. Induction of SOCS-1 is likely responsible for the hepatic inflammatory injure in the MCD fed mice. While SOCS-1 was not sufficient enough to inhibit the inflammatory injure that have been induced by MCD [31]. Up-regulation of HO-1 by hemin and Ad-HO-1 could improve the hepatic inflammation. The expression of SOCS-1 was correlated with the improved hepatic inflammatory injury. This anti-inflammatory effect might due to the decreased expression of hepatic TNF-α and IL-6 mRNA. In keeping with our findings, Wei et al. [24] reported that exposure of the liver to carbon tetrachloride (CCl4) was associated with inflammatory cell infiltration, which was attenuated in hemin-pretreated CCl4-exposed animals. Furthermore, Kim et al. found that induction of HO-1 decreased TNF-α expression and reduced adipogenesis in Zucker rats [32]. Thus, a reduction of inflammation induced by HO-1 achieved by inhibiting release of the inflammatory mediators, finally relieved hepatic inflammatory damage and fibrosis.

Following sustained oxidative stress and inflammatory injury, HSCs become activated and transdifferentiate into myofibroblasts [33]. The activation of HSCs, a key issue in the pathogenesis of hepatic fibrosis [34], is mediated by various cytokines and ROS released from the damaged hepatocytes and activated Kupffer cells. Moreover, HSCs are the primary target cells for inflammatory stimuli [35]. In this study, the mRNA and protein expression of α-SMA, a biomarker of activated HSCs, showed a prominent increase in the livers of MCD diet mice, indicating an increase of stellate cell activation. Administration of hemin or Ad-HO-1 could reduce the expression of α-SMA, suggesting that the HSCs activation was inhibited by HO-1. It has been shown that induction of HO-1 by hemin blunted hepatic myofibroblasts proliferation and procollagen I mRNA expression, which implicated that HO-1 was a major antifibrogenic pathway in human hepatic myofibroblasts [36]. To clarify the mechanism by which the MCD diet caused hepatic fibrosis in mice, the expression of TGF-β1, one of the central fibrogenic factors involved in the process of myofibroblast activation and collagen synthesis in the liver was estimated [37]. The mRNA and protein expression of TGF-β1 were increased in the livers of MCD fed mice, and further up-regulated in ZnPP-IX treatment mice. However, up-regulation of HO-1 by hemin or Ad-HO-1 favorably modulated the TGF-β1 expression. This effect was supported by observations that the overexpression of HO-1 could exert its inhibitory effects on the transcript activities of TGF-β1 in adeno-associated virus-mediated HO-1(rAAV/HO-1)-transduced animals [38]. On this basis, Up-regulation of HO-1 might function as a negative regulator in the control of fibrogenic activities of HSCs.

In addition to being a major source of ECM production, activated HSCs also show increased expression of MMP-2 and MMP-9, which can degrade several components of the normal liver matrix, including collagen IV, laminin, and fibronectin, and are important in the remodeling of matrix during tissue repair processes [39]. It was likely that degradation of the ECM by MMP-2 and MMP-9 altered cell-matrix and cell-cell interactions and enhanced hepatocyte susceptibility to necrosis [40], and HO-1 introduction might attenuate this process by decreasing MMP-2 and MMP-9 expression. Similar results have been obtained from the findings that the reversion of myocardial extracellular remodeling in the heart treated by Ad-hHO-1-transfected mesenchymal stem cells was also accompanied by a decrease of MMP-2 and MMP-9 expression, suggesting that HO-1 overexpression confered anti-fibrogenic property [41].

In conclusion, the present study showed that up-regulation of HO-1 provided a beneficial role in modulating oxidative stress, inflammatory insult and HSCs activation in the livers with fibrosing steatohepatitis. This effect was associated with the decreased expression of inflammatory cytokines TNF-α, IL-6, SOCS1 and pro-fibrogenic factors α-SMA, TGF-β1, MMP-2 and MMP-9. Clearly, further studies are still necessary to clarify at which level HO-1 plays the beneficial anti-inflammatory, anti-oxidative stress and anti-fibrotic effects.

## List of Abbreviations

HO-1: heme oxygenase-1; MCD: methionine-choline deficient; ALT: alanine aminotransferase; AST: aspartate aminotransferase; NASH: nonalcoholic steatohepatitis; ZnPP-IX: zinc protoporphyrin IX; HSC: hepatic stellate cell; ROS: reactive oxygen species; TNF-α: tumor necrosis factor-alpha; IL-6: interleukin-6; SOCS1: suppressor of cytokine signaling-1; α-SMA: α-smooth muscle actin; TGF-β1: transforming growth factor beta 1; MMP-2: matrix metallopeptidase-2; MMP-9: matrix metallopeptidase-9: TBARS, thiobarbituric acid reactive substances; RT-PCR: reverse transcription semi-quantitative polymerase chain reaction.

## Financial disclosures and conflicts of interest

The authors declare that they have no competing interests.

## Authors' contributions

YN and JY designed and organized the research; RW, WW, LK, FH, SZ, and LK performed the experiments; YN and RW analyzed data; YN, RW and JY wrote the paper. All authors read and approved the final manuscript.
